# Effects of resveratrol on laminar shear stress-induced mitochondrial biogenesis in human vascular endothelial cells

**DOI:** 10.20463/jenb.2019.0002

**Published:** 2019-03-31

**Authors:** Ji-Seok Kim, Joon-Young Park

**Affiliations:** 1 Department of Physical Education, Gyeongsang National University, Jinju Republic of Korea; 2 Department of Kinesiology, Temple University, Philadelphia USA; 3 Cardiovascular Research Center, Lewis Katz School of Medicine, Temple University, Philadelphia USA

**Keywords:** Resveratrol, Laminar shear stress, SIRT1, Mitochondrial biogenesis

## Abstract

**[Purpose]:**

The purpose of the study was to determine the combined effects of resveratrol supplementation with high-flow LSS on mitochondrial biogenesis in human vascular endothelial cells.

**[Methods]:**

Cultured human umbilical vein endothelial cells were treated with 20 μM of RSV. For the shear experiments, cells grown to a >90% confluence were exposed to physiological levels of LSS (5 to 20 dyne/cm2) for 12 to 36 hours using a cone and plate shear apparatus. Gene expressions were analyzed by western blotting.

**[Results]:**

Depletion of mitochondrial integrity was directly associated with increase in endothelial activation/dysfunction. The expressions of mitochondrial biogenesis regulator genes, such as SIRT1, PGC-1α, and TFAM, and the mitochondrial contents were significantly increased after treatment with both resveratrol and high-flow LSS for 12 hours. However, supplementation of resveratrol to high-flow LSS for a prolonged duration had no synergistic effect on the levels of mitochondrial biogenesis regulator gene expressions and mitochondrial content compared to the LSS treatment alone.

**[Conclusion]:**

The present study demonstrated that the supplementation of resveratrol to high-flow LSS has no synergistic effects on enhancing mitochondrial integrity in human vascular endothelial cells.

## INTRODUCTION

Mitochondria occupy a relatively small proportion of the cytoplasmic volume in vascular endothelial cells (EC) compared to other types of cells with high energy demands, such as cardiomyocytes^1^. Mitochondria are commonly described as the intracellular power plants due to their capacity to generate ATP, the energy transfer molecule. However, the role of mitochondria in vascular homeostasis exceeds oxidative phosphorylation^2^. The endothelial mitochondria are thought to play key regulatory roles in cell signaling^3^, calcium handling^4^, and cell survival^2^. Indeed, an impaired mitochondrial network in the ECs has been directly linked to the functional deterioration associated with cardiovascular diseases^5^^,^^6^.

Resveratrol (3,5,4’-trihydroxystilbene) (RSV), a natural polyphenol compound found in grape skins and red wine, is known for its calorie restriction or exercise mimetic characteristics, such as enhanced antioxidant properties and vasoprotective functions^7^^-^^10^. Therefore, the application of RSV in ECs has been accepted as an effective pharmacological treatment for the prevention and improvement of cardiovascular pathologies via sirtuin 1 (SIRT1)-dependent activation of the PGC-1α^9^^-^^12^. SIRT1, an NAD+-dependent histone deacetylase, is known to regulate cell cycle, apoptosis, cellular metabolism, and longevity^13^^,^^14^ and contribute to the maintenance of vascular function through mitochondrial biogenesis-dependent mechanisms in vascular ECs^15^^,^^16^.

Vascular EC surfaces are continuously exposed to hemodynamic shear stress, a friction force generated by blood flow, which is increased during aerobic exercise^17^^,^^18^. High-flow laminar shear stress (LSS) is a known mechanistic stimulant for the maintenance of vascular homeostasis by enhancing SIRT1/PGC-1α pathways in vascular ECs. Previous studies have shown that the application of high-flow LSS in cultured human umbilical vein endothelial cells (HUVEC) induces increase in the expression of SIRT1 as well as its downstream gene, PGC-1α, resulting in the promotion of mitochondrial biogenesis^15^^,^^16^.

Although both RSV and LSS are known to be prominent activators of mitochondrial biogenesis, the synergistic effects of RSV supplemented with exercise on mitochondrial integrity in vascular ECs are still obscure. In addition, while the effects of exercise training and enhanced LSS on cardiovascular health are evident^16^^,^^18^, the effects of RSV are still controversial^19^^,^^20^. Furthermore, while studies have made vigorous endeavors to examine the combined effects of exercise and resveratrol treatment on skeletal muscle metabolism^21^^-^^23^, the information on vascular homeostasis is still insufficient. The purpose of the current study was to determine the synergistic effects of RSV supplementation to high-flow LSS compared to the effects of LSS treatment alone on the gene expression of regulators of mitochondrial biogenesis and mitochondrial integrity in vascular ECs.

## METHODS

### Cell culture and morphological analysis

HUVEC lines from Lonza were grown in M199 medium (Cellgro, Manassas, VA) supplemented with 20% fetal bovine serum (FBS), heparine, penicillin/streptomycin, Fungizone, and endothelial cell growth supplement (ECGS) (Sigma, Saint Louis, MO), and they were maintained in 100 mm tissue culture dishes at 37°C in a 5% CO2 atmosphere. All the experiments with HUVEC were conducted between the 3^rd^ and 5^th^ passages. For some mitochondrial depletion experiments, HUVECs were treated with rotenone (2 μM, 12 h) before shear exposure.

The morphological analysis of the cultured HUVECs were performed by phase-contrast microscopy (Axiovert 40 CFL, Zeiss) after exposure to either 20 μM of RSV or 20 dyne/cm^2^ LSS.

### Resveratrol and shear stress treatment

For SIRT1 activation by pharmacological treatment, cells were treated with 20 μM of resveratrol (*trans*-3,4’,-5-trihydroxystilebene) (RSV) , an optimized concentration determined in our previous study^15^. Dimethyl sulfoxide (DMSO), which is the solvent for RSV, was used as the control.

For the shear experiments, HUVECs were grown till 90-100% confluence, the medium was replaced with shear media and exposed to the physiological levels of shear stress (5 to 20 dyne/cm^2^) for 12 to 36 hours using a cone and plate viscometer (0.5° cone angle), which was placed inside the CO2 cell culture incubator, as described previously^15^^,^^24^^,^^25^. The shear media used was M199 supplemented with 2% FBS and ECGS. All shear experiments were conducted under sterile conditions.

### Endothelial microparticles immunolabeling and flow cytometry

The level of endothelial microparticles (EMP) was determined by flow cytometry, as previously described^15^^,^^26^. Briefly, the isolated microparticle (MP) pellets were incubated with fluorochrome-labeled antibodies for 20 minutes at room temperature(20 to 25 ℃) in the dark on a shaker. The samples were diluted with 500 μl of 0.22 μm double filtered phosphate buffered saline before analysis. Samples were measured using a BD LSRII flow cytometer and BD FACSDIVA software. The activated microparticles from EC were defined as CD62E+ and with vesicles sizes of < 1 μm. A logarithmic scale was implemented for the forward scatter signals, side scatter signals and each fluorescent channel. Size calibration was done with 0.9 μm beads. Unstained samples were used to distinguish true events from noise and to increase the specificity of EMP detection for each sample. The flow rate was set at medium and all samples were run for 180 seconds. A mean sample processing rate of 101 μl/180 seconds was calculated using calibration beads. EMP counts per μl of medium were described as EMP production.

### Antibodies and western blotting

Immediately following RSV and/or LSS applications, HUVECs were harvested for protein analysis. Aliquots from cell lysates were separated by SDS-PAGE on 10% gels and transferred onto PVDF membranes, which were blocked with 5% non-fat dry milk dissolved in Tris-buffered saline and then incubated overnight with primary antibodies at 4°C. Immunoreactive proteins were detected by chemiluminescence with Thermo Scientific SuperSignal (Pierce Biotechnology, IL). The primary antibodies used included anti-SIRT1 (Cell Signaling Technology, Danvers, MA), anti-PGC-1α (NOVUS, Littleton, CO), anti-TFAM (NOVUS, Littleton, CO), anti-VDAC1/Porin (ABcam, Cambridge, MA), and anti-α-tubulin (Sigma, St Louis, MO).

### Statistical analysis

All the data presented in the figures were expressed as the means ± SE. The differences across experimental conditions were assessed by analysis of variance (one-way ANOVA) followed by post hoc testing with the Fisher’s least significant difference test. P < 0.05 was considered statistically significant for all analysis.

## RESULTS

### Mitochondrial integrity in endothelial homeostasis

**Figure 1:**
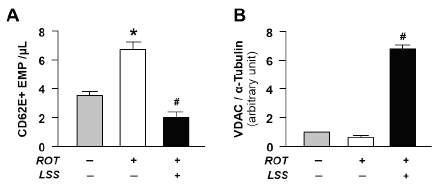
Role of mitochondrial integrity in endothelial homeostasis. A: The levels of EMP released from activated ECs. B: Mitochondrial content determined by the abundance of VDAC expression. Bar graphs display the results of densitometry analyses. The bar graphs in all the panels are mean ± SEM. ^*^, p < 0.05 compared to the static control; #, p < 0.05 compared to rotenone treatment. ROT, rotenone; LSS, laminar shear stress

First of all, to determine the importance of mitochondrial integrity in endothelial homeostasis, HUVECs were subjected to rotenone (2 μM for 12 hours), a known mitochondrial complex I inhibitor. Rotenone-induced depletion of mitochondrial integrity significantly increased the EMP production, which is a determinant of endothelial activation and dysfunction (Fig. 1A). However, LSS-induced enhancement of mitochondrial content (Fig. 1B) reversed the EMP production promoted by rotenone (Fig. 1A). These results represent that mitochondrial integrity is critical for the endothelial homeostasis.

### Effects of high-flow laminar shear stress on mitochondrial content 

**Figure 2:**
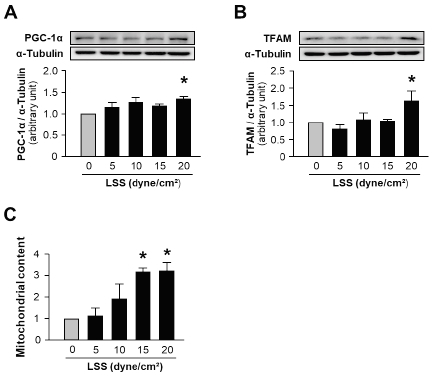
Effects of high-flow laminar shear stress on mitochondrial biogenesis in human vascular endothelial cells. A: PGC-1α expression after 12 hours of treatment with LSS of different magnitudes. B: TFAM expression after 12 hours of LSS treatment. C: Mitochondrial content determined by the abundance of VDAC. HUVECs were exposed to LSS at 5 to 20 dyne/cm2 for 12 hours. 0 dyne/cm^2^ indicates static control. The bar graphs display the results of densitometry analyses. The bar graphs in all panels are mean ± SEM. ^*^, p < 0.05 compared to the static control

Next, in order to examine the effects of LSS on mitochondrial biogenesis, we subjected confluent monolayers of human ECs to different magnitudes of LSS (0, 5, 10, 15, and 20 dyne/cm^2^) for 12 hours. The key regulator genes for mitochondrial biogenesis, such as PGC-1α and TFAM, were significantly increased by a magnitude of 20 dyne/cm2, denoting high-flow LSS (Fig. 2A and 2B). The mitochondrial content, determined by the abundance of mitochondrial outer membrane protein, VDAC, was also increased by high-flow LSS (15 to 20 dyne/cm^2^) corresponding to the expression of mitochondrial biogenesis regulator genes (Fig. 2C). These results confirm that the high-flow LSS, which is increased by exercise, has direct effects on mitochondrial biogenesis.

### Effects of resveratrol and laminar shear stress on cell morphological change and SIRT1 expression

**Figure 3:**
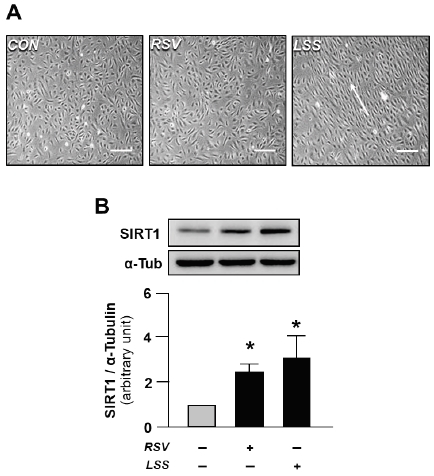
Effects of resveratrol and laminar shear stress on cell morphology and SIRT1 expressions. A: Morphological changes in the HUVECs exposed to either 20 μM of RSV or 20 dyne/cm2 LSS for 12 hours. B: SIRT1 expressions. Arrow indicates the direction of shear flow. Scale bars indicate 100 μm. The bar graphs display the results of densitometry analysis. The bar graphs in the panel are mean ± SEM. ^*^, p < 0.05 vs. static control. CON, static control; RSV, resveratrol; LSS, laminar shear stress

In static states, vascular ECs have cobblestone shaped morphology, which is a typical characteristic of human vascular ECs. RSV treatment could not induce morphological changes in the HUVECs. However, the exposure to the high-flow LSS induced an elongation of ECs in the direction of the flow (Fig. 3A). Hence, both RSV and LSS treatments enhanced the level of SIRT1, which is the key regulator gene for increasing expression level of for induction of mitochondrial biogenesis factors, such as PGC-1α and TFAM (Fig. 3B). These results represent that although there were differences in morphological changes induced by RSV and LSS treatments, both of these applications are potent stimulants for the upregulation of mitochondrial biogenesis regulator expression.

### The synergistic effect of resveratrol supplementation with high-flow laminar shear stress on mitochondrial biogenesis 

**Figure 4:**
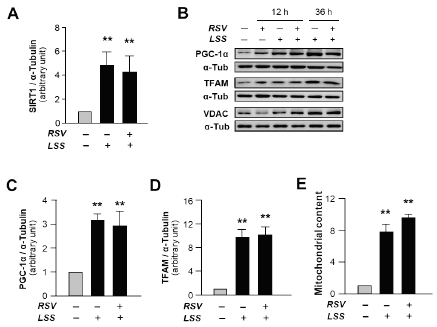
Lack of synergistic effects of resveratrol supplementation to high-flow laminar shear stress on mitochondrial biogenesis. A: SIRT1 expression after treatment with high-flow LSS for 36 hours or a combination of RSV and LSS. B: Representative gene expressions analyzed by western blotting . C: PGC-1α and D: TFAM expression levels and E: mitochondrial content determined by the abundance of VDAC expression after treatment with high-flow LSS for 36 hours or a combination of RSV and LSS. The bar graphs display the results of densitometry analyses. The bar graphs in all panels are mean ± SEM. ^**^, p < 0.01 compared to static control. RSV, resveratrol; LSS, laminar shear stress

To examine the synergistic effect of RSV supplemented with high-flow LSS on the expressions of SIRT1 and mitochondrial biogenesis regulators, as well as mitochondrial contents, compared to the solitary treatment of LSS, confluent monolayers of HUVECs were subjected to either 20 dyne/cm^2^ LSS treatment alone or in combination with 20 μM of RSV for 36 hours. The expressions of SIRT1 (Fig. 4A), PGC-1α (Fig. 4C), and TFAM (Fig. 4D) were significantly increased by both LSS and the combined treatment of RSV and LSS. Along with mitochondrial biogenesis regulator gene expressions, the mitochondrial content levels were also significantly increased by both LSS and combined treatment (Fig. 4E). However, there were no obvious synergistic effects of the combined treatment of RSV and LSS compared to the solitary treatment of LSS on mitochondrial biogenesis (Fig. 4A-4E). These results show that the effects of LSS dominates the effects of RSV on endothelial mitochondrial biogenesis .

## DISCUSSION

Mitochondria found in endothelial cells play key regulatory roles in cell signaling and survival^2^. Mitochondrial damage and depletion are seen in many facets of vascular disease development including atherosclerosis with the impairment of endothelial viability. Various pharmacological inhibitors of mitochondrial metabolism, used for treatment, are known stimulants that can cause impairment of endothelium-dependent vasodilatory function and increase in mitochondrial ROS production^27^^,^^28^. Rotenone, an inhibitor of the mitochondrial complex I, abolishes acetylcholine-induced vascular relaxation^29^. In our study, first, we confirmed that in ECs subjected to rotenone, depletion of mitochondrial integrity is induced and EMP production is increased. These are conventionally accepted determinants of endothelial activation and dysfunction^30^^-^^32^. This result confirms that the mitochondrial integrity is critical for the endothelial homeostasis.

In a number of previous studies, the beneficial effects of RSV on mitochondrial biogenesis and endothelial protection have been introduced^33^^,^^34^. In vascular ECs, the effects of high-flow LSS on the maintenance of vascular homeostasis via induction of mitochondrial biogenesis^16^, modulation of endothelial activation^35^, and attenuation of apoptosis^36^ are evident. However, despite the fact that both RSV and LSS are potent stimulants of vascular health, the synergistic effects of RSV supplemented with exercise-induced high-flow LSS on mitochondrial integrity in ECs are still unclear. In a recently published in vivo study, the supplementation of RSV in physically inactive aged men diminished the positive effects of exercise training on cardiovascular functions, such as blood pressure and maximal oxygen capacity^19^. However, the results of this study are still controversial as opposing claims have been made by numerous previous studies^20^. In this regard, our present study clarifies this uncertainty by confirming the effects of RSV supplementation to the high-flow LSS. The major finding of the present study is that the both RSV treatment and high-flow LSS application in cultured human vascular ECs increases the expression levels of regulators of mitochondrial biogenesis as well as the mitochondrial contents. However, concomitant supplementation of RSV to high-flow LSS has no synergistic effects on enhancing mitochondrial integrity, compared to the LSS application alone.

Consistent exposure of ECs to high-flow LSS induces changes in EC morphology as seen by the polygonal shape of ECs due to elongation of cells following the flow direction^18^. In this study, we also demonstrated that the consistent exposure of ECs to high-flow LSS transformed their morphologies from cobblestone shaped, which is the typical morphology of static state ECs, into fusiform shape, in order to follow the flow direction. However, the combined treatment of RSV and LSS did not elicit any distinguishable changes in the cell morphologies, compared to the solitary treatment of LSS.

In the recent study mentioned above, supplementation of RSV to exercise training had no effect on SIRT1 expression^19^. Despite the in vitro experimental conditions used in our current study, we observed that SIRT1 expression was significantly increased by treatments with both RSV and LSS in vascular ECs. SIRT1 is a master regulator of stress response and energy response mediating expression of PGC-1α, a mitochondrial biogenesis factor^16^. It has been seen that treatment of human vascular ECs with RSV, a SIRT1 activator, increased mitochondrial mass and PGC-1α expression^33^, and the inhibition of SIRT1 in rat arteries attenuated vasodilatory function^37^. In our previous study, we have also demonstrated increase SIRT1 and SIRT1-regulated gene expression of regulators such as PGC-1α and TFAM in cultured human vascular ECs exposed to RSV^15^. However, interestingly, in the present study, synergistic effects of RSV supplementation to high-flow LSS on the expressions of SIRT1 and mitochondrial biogenesis regulator genes, compared to LSS treatment alone, were not observed. Also, we did not observe any further increase of mitochondrial integrity in the cells that have undergone combined treatment. Taken together, RSV supplementation exerts its effects on vascular health via SIRT1-dependent mechanism. However, when it is applied with a high-flow LSS, a distinctly enhanced effect on endothelial mitochondrial integrity compared to LSS alone is not observed.

## CONCLUSION

The present study demonstrates a critical role of LSS in enhancing endothelial mitochondrial integrity, which is crucial for preserving endothelial homeostasis. However, the supplementation of RSV, which is known for its exercise mimetic characteristics in vascular ECs, to high-flow LSS does not have synergistic effects on mitochondrial biogenesis compared to the LSS treatment alone. In conclusion, although RSV is beneficial for vascular health and mimics effects of exercise, high-flow LSS, which is systemically elevated during aerobic exercise in the vessel wall, dominates the effects of RSV as seen in the combined treatments.

## References

[1] Oldendorf WH, Cornford ME, Brown WJ. (1977). The large apparent work capability of the blood-brain barrier: a study of the mitochondrial content of capillary endothelial cells in brain and other tissues of the rat. *Ann Neurol*.

[2] Yu E, Mercer J, Bennett M. (2012). Mitochondria in vascular disease. *Cardiovasc Res*.

[3] Quintero M, Colombo SL, Godfrey A, Moncada S. (2006). Mitochondria as signaling organelles in the vascular endothelium. *Proc Natl Acad Sci U S A*.

[4] Szabadkai G, Duchen MR. (2008). Mitochondria: the hub of cellular Ca2+ signaling. *Physiology (Bethesda)*.

[5] Kluge MA, Fetterman JL, Vita JA. (2013). Mitochondria and endothelial function. *Circ Res*.

[6] Kizhakekuttu TJ, Wang J, Dharmashankar K, Ying R, Gutterman DD, Vita JA, Widlansky ME. (2012). Adverse alterations in mitochondrial function contribute to type 2 diabetes mellitus-related endothelial dysfunction in humans. *Arterioscler Thromb Vasc Biol*.

[7] Schmitt CA, Heiss EH, Dirsch VM. (2010). Effect of resveratrol on endothelial cell function: Molecular mechanisms. *Biofactors*.

[8] Pervaiz S, Holme AL. (2009). Resveratrol: its biologic targets and functional activity. *Antioxid Redox Signal*.

[9] Baur JA, Pearson KJ, Price NL, Jamieson HA, Lerin C, Kalra A, Prabhu VV, Allard JS, Lopez-Lluch G, Lewis K, Pistell PJ, Poosala S, Becker KG, Boss O, Gwinn D, Wang M, Ramaswamy S, Fishbein KW, Spencer RG, Lakatta EG, Le Couteur D, Shaw RJ, Navas P, Puigserver P, Ingram DK, de Cabo R, Sinclair DA. (2006). Resveratrol improves health and survival of mice on a high-calorie diet. *Nature*.

[10] Lagouge M, Argmann C, Gerhart-Hines Z, Meziane H, Lerin C, Daussin F, Messadeq N, Milne J, Lambert P, Elliott P, Geny B, Laakso M, Puigserver P, Auwerx J. (2006). Resveratrol improves mitochondrial function and protects against metabolic disease by activating SIRT1 and PGC-1alpha. *Cell*.

[11] Yap S, Qin C, Woodman OL. (2010). Effects of resveratrol and flavonols on cardiovascular function: Physiological mechanisms. *Biofactors*.

[12] Ungvari Z, Sonntag WE, de Cabo R, Baur JA, Csiszar A. (2011). Mitochondrial protection by resveratrol. *Exerc Sport Sci Rev*.

[13] Borradaile NM, Pickering JG. (2009). NAD(+), sirtuins, and cardiovascular disease. *Curr Pharm Des*.

[14] Blander G, Guarente L. (2004). The Sir2 family of protein deacetylases. *Annu Rev Biochem*.

[15] Kim JS, Kim B, Lee H, Thakkar S, Babbitt DM, Eguchi S, Brown MD, Park JY. (2015). Shear stress-induced mitochondrial biogenesis decreases the release of microparticles from endothelial cells. *Am J Physiol Heart Circ Physiol*.

[16] Chen Z, Peng IC, Cui X, Li YS, Chien S, Shyy JY. (2010). Shear stress, SIRT1, and vascular homeostasis. *Proc Natl Acad Sci U S A*.

[17] Cunningham KS, Gotlieb AI. (2005). The role of shear stress in the pathogenesis of atherosclerosis. *Lab Invest*.

[18] Malek AM, Alper SL, Izumo S. (1999). Hemodynamic shear stress and its role in atherosclerosis. *JAMA*.

[19] Gliemann L, Schmidt JF, Olesen J, Biensø RS, Peronard SL, Grandjean SU, Mortensen SP, Nyberg M, Bangsbo J, Pilegaard H, Hellsten Y. (2013). Resveratrol blunts the positive effects of exercise training on cardiovascular health in aged men. *J Physiol*.

[20] Buford TW, Anton SD. (2014). Resveratrol as a supplement to exercise training: friend or foe?. *J Physiol*.

[21] Olesen J, Gliemann L, Biensø R, Schmidt J, Hellsten Y, Pilegaard H. (2014). Exercise training, but not resveratrol, improves metabolic and inflammatory status in skeletal muscle of aged men. *J Physiol*.

[22] Gliemann L, Olesen J, Biensø RS, Schmidt JF, Akerstrom T, Nyberg M, Lindqvist A, Bangsbo J, Hellsten Y. (2014). Resveratrol modulates the angiogenic response to exercise training in skeletal muscles of aged men. *Am J Physiol Heart Circ Physiol*.

[23] Menzies KJ, Singh K, Saleem A, Hood DA. (2013). Sirtuin 1-mediated effects of exercise and resveratrol on mitochondrial biogenesis. *J Biol Chem*.

[24] Park JY, Farrance IK, Fenty NM, Hagberg JM, Roth SM, Mosser DM, Wang MQ, Jo H, Okazaki T, Brant SR, Brown MD. (2007). NFKB1 promoter variation implicates shear-induced NOS3 gene expression and endothelial function in prehypertensives and stage I hypertensives. *Am J Physiol Heart Circ Physiol*.

[25] Kim B, Lee H, Kawata K, Park JY. (2014). Exercise-mediated wall shear stress increases mitochondrial biogenesis in vascular endothelium. *PloS One*.

[26] Brown MD, Feairheller DL, Thakkar S, Veerabhadrappa P, Park JY. (2011). Racial differences in tumor necrosis factor-alpha-induced endothelial microparticles and interleukin-6 production. *Vasc Health Risk Manag*.

[27] Boveris A, Chance B. (1973). The mitochondrial generation of hydrogen peroxide. General properties and effect of hyperbaric oxygen. *Biochem J*.

[28] Dionisi O, Galeotti T, Terranova T, Azzi A. (1975). Superoxide radicals and hydrogen peroxide formation in mitochondria from normal and neoplastic tissues. *Biochim Biophys Acta*.

[29] Csiszar A, Labinskyy N, Orosz Z, Ungvari Z. (2006). Altered mitochondrial energy metabolism may play a role in vascular aging. *Med Hypotheses*.

[30] Boulanger CM, Amabile N, Tedgui A. (2006). Circulating microparticles: a potential prognostic marker for atherosclerotic vascular disease. *Hypertension*.

[31] Burger D, Schock S, Thompson CS, Montezano AC, Hakim AM, Touyz RM. (2013). Microparticles: biomarkers and beyond. *Clin Sci (Lond)*.

[32] Jenkins NT, Padilla J, Boyle LJ, Credeur DP, Laughlin MH, Fadel PJ. (2013). Disturbed Blood Flow Acutely Induces Activation and Apoptosis of the Human Vascular Endothelium. *Hypertension*.

[33] Csiszar A, Labinskyy N, Pinto JT, Ballabh P, Zhang H, Losonczy G, Pearson K, de Cabo R, Pacher P, Zhang C, Ungvari Z. (2009). Resveratrol induces mitochondrial biogenesis in endothelial cells. *Am J Physiol Heart Circ Physiol*.

[34] Ungvari Z, Bagi Z, Feher A, Recchia FA, Sonntag WE, Pearson K, de Cabo R, Csiszar A. (2010). Resveratrol confers endothelial protection via activation of the antioxidant transcription factor Nrf2. *Am J Physiol Heart Circ Physiol*.

[35] Ando J, Tsuboi H, Korenaga R, Takada Y, Toyama-Sorimachi N, Miyasaka M, Kamiya A. (1994). Shear stress inhibits adhesion of cultured mouse endothelial cells to lymphocytes by downregulating VCAM-1 expression. *Am J Physiol*.

[36] Dimmeler S, Haendeler J, Rippmann V, Nehls M, Zeiher AM. (1996). Shear stress inhibits apoptosis of human endothelial cells. *FEBS Lett*.

[37] Mattagajasingh I, Kim CS, Naqvi A, Yamamori T, Hoffman TA, Jung SB, DeRicco J, Kasuno K, Irani K. (2007). SIRT1 promotes endothelium-dependent vascular relaxation by activating endothelial nitric oxide synthase. *Proc Natl Acad Sci U S A*.

